# Dephasing Dynamics in a Non-Equilibrium Fluctuating Environment

**DOI:** 10.3390/e25040634

**Published:** 2023-04-08

**Authors:** Xiangjia Meng, Yaxin Sun, Qinglong Wang, Jing Ren, Xiangji Cai, Artur Czerwinski

**Affiliations:** 1 School of Information Engineering, Shandong Youth University of Political Science, Jinan 250103, China; 2 New Technology Research and Development Center of Intelligent Information Controlling in Universities of Shandong, Shandong Youth University of Political Science, Jinan 250103, China; 3 School of Science, Shandong Jianzhu University, Jinan 250101, China; 4 Institute of Physics, Faculty of Physics, Astronomy and Intypeatics, Nicolaus Copernicus University in Torun, ul. Grudziadzka 5, 87-100 Torun, Poland

**Keywords:** open quantum systems, decoherence, non-equilibrium environmental fluctuations

## Abstract

We performed a theoretical study of the dephasing dynamics of a quantum two-state system under the influences of a non-equilibrium fluctuating environment. The effect of the environmental non-equilibrium fluctuations on the quantum system is described by a generalized random telegraph noise (RTN) process, of which the statistical properties are both non-stationary and non-Markovian. Due to the time-homogeneous property in the master equations for the multi-time probability distribution, the decoherence factor induced by the generalized RTN with a modulatable-type memory kernel can be exactly derived by means of a closed fourth-order differential equation with respect to time. In some special limit cases, the decoherence factor recovers to the expression of the previous ones. We analyzed in detail the environmental effect of memory modulation in the dynamical dephasing in four types of dynamics regimes. The results showed that the dynamical dephasing of the quantum system and the conversion between the Markovian and non-Markovian characters in the dephasing dynamics under the influence of the generalized RTN can be effectively modulated via the environmental memory kernel.

## 1. Introduction

Quantum coherence is an important phenomenon in the microcosmic world, which has been attracting continuous attention with the advance of experimental technologies. In a wide variety of applications related to quantum physics, the destruction of coherence is inevitable owing to the reason that any quantum system keeps interacting with the surrounding environments. The unavoidable interactions of an open quantum system with its surroundings bring about its correlations with environmental states and make the system lose coherence in dynamical evolution [1,2,3,4,5,6]. The loss of the quantum coherence of open systems induced by the environments is usually called decoherence, which is widely used to describe the quantum–classical transition and is regarded as a great obstacle to the design and realization of experimental devices for quantum information processing. Recently, the investigations of the decoherence process of open quantum systems have received more and more considerable attention, which plays a significant role in a series of essential issues in quantum information science, such as quantum computation, quantum measurement, quantum control, and so on [7,8,9,10,11,12,13,14,15,16,17,18,19,20,21].

Over the past several decades, the quantum decoherence dynamics of open systems has been investigated by making the assumption that system–environment coupling is weak and by ignoring the memory effect of the actual dynamical evolution. These treatments are usually called Markovian approximations, and the quantum dynamics of open systems is generally described in the Lindblad-type master equations. However, the couplings with the environment are not weak, and the quantum evolution of the open system displays a memory effect in the vast majority of realistic cases. In these situations, the Markovian approximations are no longer valid, and the non-Markovian character exhibited in the decoherence dynamics plays a non-negligible role [22,23,24,25]. Under the influence of environments exhibiting equilibrium fluctuations, the study of non-Markovian quantum dynamics has drawn increasing attention by treating the environmental noise with a stationary statistical property [13,20,26,27,28,29,30,31,32,33,34,35,36,37,38,39,40,41,42,43,44,45,46]. Recently, it was shown that the non-equilibrium environmental fluctuations become dominant in some transient and ultra-fast physical or biological processes. The instantaneous environmental state influenced by the initial couplings to the system cannot return to equilibrium rapidly, corresponding to the statistics of the environmental noise no longer being stationary [47,48]. Thus, to study quantum dynamics in these situations, the effects of non-equilibrium environmental fluctuations should be taken into full consideration.

Random telegraph noise (RTN) as the widely used classical noise with non-Gaussianity has been the subject of the theoretical simulation of the influences of environmental fluctuations on open quantum systems [49,50,51,52,53,54,55,56,57,58,59]. In some previous research, the environmental fluctuations governed by the RTN were usually assumed to have stationary and Markovian statistical properties. Actually, this assumption is just an idealization of the environmental fluctuations in statistics. In some realistic situations, the statistical properties of the fluctuating environments may be non-stationary and non-Markovian. On the basis of this fact, the non-Markovian RTN governed by an exponential-type memory kernel with stationary and non-stationary statistics was proposed and discussed in succession. The generalized RTN with non-stationary and non-Markovian statistics has been employed extensively to investigate the related questions concerning the quantum decoherence dynamics of open systems in the presence of non-equilibrium environmental fluctuations [60,61,62,63,64,65,66,67]. In recent research, the stationary RTN with non-Markovian statistics governed by a memory kernel of a modulatable-type has also been put forward. It has been demonstrated that the dynamical dephasing of the quantum two-state system can be modulated by the environmental memory kernel in an equilibrium environment [68]. The exact expression for the decoherence factor for open quantum systems in the presence of generalized RTN with non-stationary and non-Markovian statistics is rather difficult to obtain. It is shown that the decoherence factor satisfies a time differential equation of third-order under the influence of the generalized RTN with an exponential-type memory kernel [61]. However, in a non-equilibrium environment governed by the generalized RTN with a modulatable-type memory kernel, the decoherence factor of a quantum two-state system has not been derived. The environmental effect of memory modulation in the dynamical dephasing in a non-equilibrium environment has not been investigated yet. Therefore, there are some important physical issues arising naturally and that we should further address. Under the influence of the generalized RTN with a modulatable memory kernel, is it possible to derive the decoherence factor exactly by establishing a closed differential equation with respect to time? How do the memory effects of the generalized RTN modulate the quantum dynamical dephasing of the system in a non-equilibrium fluctuating environment? Can we convert the Markovian and non-Markovian characters in the dephasing dynamics by changing the modulation frequency in the memory kernel of the generalized RTN?

In the present paper, we theoretically investigated the dephasing dynamics of a quantum two-state system under the influence of a fluctuating environment displaying non-equilibrium fluctuations described by the generalized RTN with non-stationary and non-Markovian statistics. The decoherence factor satisfies a closed fourth-order time differential equation under the generalized RTN with a modulatable-type memory kernel. The expression of the decoherence factor can be exactly simplified as the previous ones in some special limit cases of the environmental memory kernel. We analyzed the environmental effect of the memory modulation in the dynamical dephasing in four types of dynamics regimes: weak coupling weak memory regime, weak coupling strong memory regime, strong coupling weak memory regime, and strong coupling strong memory regime, respectively. The results display that the quantum dephasing dynamics of the system and the conversion between the Markovian and non-Markovian characters in the dynamical dephasing can be effectively modulated via the environmental memory kernel. In addition, the boundary in the dephasing dynamics between the Markovian and non-Markovian characters is determined by the combined effects of the system–environment coupling, the environmental memory, and the environmental modulation.

The organization of the paper is as follows. We first present the theoretical framework, in Section 2, of the quantum dephasing dynamics under the influence of non-equilibrium environmental fluctuations. We derived the decoherence factor of the quantum system exactly under the generalized RTN with a modulatable-type memory kernel by establishing a closed differential equation with respect to time. In Section 3, we give the results of the quantum dynamical dephasing in four types of dynamics regimes and the dynamical conversion between the Markovian and non-Markovian characters. Finally, we give the concluding remarks in Section 4.

## 2. Quantum Dephasing under the Influence of Non-Equilibrium Environmental Fluctuations

The physical model we considered here is a quantum two-state system in interaction with a classical fluctuating environment, which displays non-equilibrium fluctuations. We assumed the environmental effects do not lead to population transfer and the quantum system undergoes pure dephasing during its dynamical evolution. The influences of the environment on the system cause the energy gap between the two states in the type $E_{1}\left(t\right)-E_{2}\left(t\right)=\hbar \omega \left(t\right)$, where $E_{k}\left(t\right)$ (k=1,2) denotes the instantaneous energy of the state *k* and ω(t) is the transition frequency between the two states |1〉 and |2〉, which fluctuates stochastically due to the coupling between the system and environment [47,48,69,70].

In terms of the spectral diffusion framework of Kubo–Anderson, the instantaneous frequency difference of the quantum system can be rewritten as $\omega \left(t\right)=\omega_{0}+\zeta \left(t\right)$, with $\omega_{0}$ denoting the standard frequency difference and ζ(t) the fluctuation part arising from the environmental effects generally governed by a classical stochastic process. Stochastic processes with a stationary statistical property have been widely used to describe the equilibrium environmental fluctuations [71]. Under the influence of the environments exhibiting non-equilibrium fluctuations, the fluctuation part ζ(t) in the instantaneous frequency difference is generally governed by a stochastic process with non-stationary statistics, which corresponds, in the physical description, to environmentally excited phonons with sharply defined phases initially [47,48].

For the quantum system prepared in an initial coherent state with the superposition of |2〉 and |1〉, the non-diagonal element in the density matrix quantifies the time-dependent coherence of the system:

(1) $$\rho_{21}\left(t\right)=D\left(t\right)e^{i\omega_{0}t}\rho_{21}\left(0\right),$$

where D(t) represents the decoherence factor, which can be written in terms of the moments of the fluctuation part ζ(t) in the Dyson series expansion:

(2) $$D\left(t\right)=\langle \exp\left[i\int_{0}^{t}dt^{\prime }\zeta \left(t^{\prime }\right)\right]\rangle =1+\sum_{n=1}^{\infty }i^{n}\int_{0}^{t}dt_{1}\cdots \int_{0}^{t_{n-1}}dt_{n}\langle \zeta \left(t_{1}\right)\cdots \zeta \left(t_{n}\right)\rangle ,$$

where 〈⋯〉 represents a statistical average taken over ζ(t). The decoherence factor D(t) closely depends on the statistical properties of the stochastic fluctuations induced by the environment. Under the influence of non-equilibrium fluctuating environments, the decoherence factor D(t) is no longer real, but complex in time, resulting from the non-stationary statistics of the fluctuation part ζ(t).

For the dynamical dephasing process of the system in a non-equilibrium fluctuating environment, there are two important physical qualities, namely the frequency shift s(t) and the dephasing rate γ(t), linked to the decoherence factor D(t), with the definitions as

(3) s(t)=−ImdD(t)/dtD(t),γ(t)=−RedD(t)/dtD(t).

The frequency shift s(t) expressed in Equation (3) can be used to distinguish the stationary and non-stationary statistics of the environmental noise between equilibrium and non-equilibrium fluctuating environments. In general, there will not appear a frequency shift for the environments exhibiting equilibrium fluctuations, whereas under the influence of non-equilibrium environmental fluctuations, the frequency shift is time-dependent. The decoherence rate γ(t) of the dephasing dynamics in Equation (3) is linked to the information exchange that takes place between the system and the environment. There is a one-way continuous information flow to the environment out of the system without environmental coherence back-action for the case that the decoherence rate γ(t) is positive at all times. For the case that γ(t) sometimes takes negative values, the information flows back into the system from the environment with the emergence of the environmental coherence back-action. According to the definition of Breuer–Laine–Piilo, the non-Markovianity, namely the total of the maximum flow of the environmental information backward to the quantum system, is written as [72]:

(4) $$N=-\int_{\gamma (t)<0}\gamma \left(t\right)|D\left(t\right)|dt=\sum_{j=1}^{\infty }|D\left(t_{2j}\right)|-|D\left(t_{1j}\right)|,$$

where $\left[t_{1j},t_{2j}\right]$ are the *j*th time intervals in which |D(t)| increases.

Combined with the expansion in the Dyson series on the basis of the moments of Equation (2), it is also possible to expand the decoherence factor D(t) by means of the cumulants of the fluctuation part ζ(t) [71]. Because both expansions involve environmental correlations of order tending to infinity, therefore, it is difficult to obtain the exact expression for the decoherence factor based on them. For the general case, we need to truncate the environmental correlations to some finite order to derive the decoherence factor approximately. Some approaches have been developed to derive the decoherence factor of a quantum two-state system under the influence of environmental noise exactly. The exact expression of the decoherence factor governed by environmental fluctuations with stationary and Markovian statistical properties can be obtained, for example, by means of the stochastic Liouville equation [73]. There are, however, very few physical models for which the decoherence factor can be exactly achieved under the influence of non-equilibrium environmental fluctuations with non-stationary and non-Markovian statistics. In the following, we derive the exact expression of the decoherence factor of the quantum two-state system under the influence of the generalized RTN by means of establishing a closed time differential equation of the decoherence factor.

### 2.1. Non-Equilibrium Environmental Fluctuations Described by Generalized RTN

It should be noted that the standard RTN is a classical stochastic process with time-homogeneity and non-Gaussianity. The standard RTN transits stochastically between the values ±1 with a mean transition rate λ and the amplitude ν in stationary and Markovian statistics [74,75,76]. The ratio of the amplitude ν to the rate λ of the transition is used to identify the weak-coupling (ν/λ<1) and strong-coupling (ν/λ>1) regimes, respectively [75,76].

It is possible to extract the characteristics of the generalized RTN with non-Markovian and non-stationary statistics from that of the standard RTN according to the classical theory of probability. The non-Markovian statistics of the generalized RTN is characterized by the master equations for the multi-time probability distributions [60]:

(5) $$\frac{\partial }{\partial t}P\left(\zeta ,t;\zeta_{1},t_{1};\cdots ;\zeta_{n},t_{n}\right)=\int_{t_{1}}^{t}K\left(t-\tau \right)\lambda TP\left(\zeta ,t;\zeta_{1},t_{1};\cdots ;\zeta_{n},t_{n}\right)d\tau ,$$

with K(t−τ) being the memory kernel of the generalized RTN and the multi-time probability $P(\zeta ,t;\zeta_{1},t_{1};\cdots ;\zeta_{n},t_{n})$ and the matrix T for transition respectively written as

(6) $$P\left(\zeta ,t;\zeta_{1},t_{1};\cdots ;\zeta_{n},t_{n}\right)=\left(\begin{array}{c} P(+\nu ,t;\zeta_{1},t_{1};\cdots ;\zeta_{n},t_{n})\\ P(-\nu ,t;\zeta_{1},t_{1};\cdots ;\zeta_{n},t_{n}) \end{array}\right),\;T=\left(\begin{array}{cc} -1 & 1\\ 1 & -1 \end{array}\right).$$

The statistical property of the environmental noise depends on its prior history because of the fact that the memory effect has been taken into consideration. The non-stationary environmental statistical property of the generalized RTN arises from the single-point probability distribution [77]:

(7) $$\begin{array}{c} P\left(\zeta ,t\right)=\frac{1}{2}\left[1+aP\left(t\right)\right]\delta_{\zeta ,\nu }+\frac{1}{2}\left[1-aP\left(t\right)\right]\delta_{\zeta ,-\nu }. \end{array}$$

where *a* is the non-stationary parameter with |a|≤1 and $P\left(t-t^{\prime }\right)=L^{-1}\left[e^{-zt^{\prime }}P\left(z\right)\right]$ denotes the auxiliary function with P(z)=1/[z+2λK(z)] and $L^{-1}$ representing the inverse Laplace transform. For the memoryless case, namely K(t−τ)=δ(t−τ), then the generalized RTN returns to the Markovian one. For the special case a=0, the generalized RTN recovers to the stationary one, which corresponds to the environmental fluctuations displaying the equilibrium feature [61,62].

Based on the statistical properties given above and on the basis of Bayes’ rule in classical probability theory, the statistical features of the generalized RTN are represented in terms of the moments of first- and second-orders:

(8) $$\begin{array}{cc} M_{1}\left(t\right) & =\langle \zeta (t)\rangle =a\nu P(t),\\ M_{2}\left(t,t^{\prime }\right) & =\langle \zeta \left(t\right)\zeta \left(t^{\prime }\right)\rangle =\nu^{2}P\left(t-t^{\prime }\right), \end{array}$$

and the factorization for the higher-order moments [61,62]:

(9) $$M_{n}\left(t_{1},t_{2},\cdots ,t_{n}\right)=P\left(t_{1}-t_{2}\right)M_{n-2}\left(t_{3},t_{4}\cdots ,t_{n}\right),$$

for the ordered time instants $t_{1}>t_{2}>\cdots >t_{n}$(n≥3). Obviously, the statistical features of the generalized RTN are closely linked to the auxiliary probability function $P(t-t^{\prime })$. Thus, we can gain all the information of the generalized RTN once we obtain the expression of the auxiliary probability function in theory.

### 2.2. Closed Dynamical Equation for the Decoherence Factor under Generalized RTN with a Modulatable Memory Kernel

In general, the type of environmental memory kernel in Equation (5) can be arbitrary. There are many types of environmental memory kernels, the exponential type, the modulatable type, the power law type, and so on [78,79,80,81,82]. The generalized RTN governed by the non-Markovian non-stationary statistical properties with an exponential memory kernel has been proposed [60,62]. It has been shown that the decoherence factor obeys a closed time differential equation of third-order in a non-equilibrium environment under the influence of the generalized RTN governed by an exponential-type memory kernel by means of the differential relations of the moments with respect to time [61,62].

We considered here the case that the type of the memory kernel in Equation (5) of the generalized RTN is a modulatable one:

(10) $$K\left(t-\tau \right)=\kappa \cos\left[\Omega \left(t-\tau \right)\right]e^{-\kappa (t-\tau )},$$

where κ is the environmental memory decay rate and Ω denotes the memory modulation frequency [80,81]. Physically, this corresponds to a model with the environmental modulation of the memory effect. In the case with the modulation frequency Ω=0, the type of environmental memory kernel becomes an exponential one. The smaller κ is, the stronger the memory effect of the generalized RTN is. In the case with the decay rate κ→+∞, the generalized RTN becomes memoryless, namely K(t−τ)=δ(t−τ), and it only displays Markovian statistics.

According to the previous work in [61,62], the dynamical equation for the decoherence factor is closely linked to the time differential relationships of the moments of the generalized RTN. Because of the fact that the ancillary probability function is related to the statistical features of the generalized RTN as in Equation (9), a closed time differential equation for the decoherence factor of the quantum system can be derived in terms of the differential relation of the auxiliary probability functional P(t). The type of memory kernel implies that the auxiliary probability function P(t) of environmental noise satisfies a closed time differential equation of third-order as follows:

(11) $$\frac{d^{3}}{dt^{3}}P\left(t\right)+c_{2}\frac{d^{2}}{dt^{2}}P\left(t\right)+c_{1}\frac{d}{dt}P\left(t\right)+c_{0}P\left(t\right)=0,$$

with the coefficients $c_{2}=2\kappa$, $c_{1}=\kappa^{2}+\Omega^{2}+2\kappa \lambda$, and $c_{0}=2\kappa^{2}\lambda$ and the initial conditions P(0)=1, (d/dt)P(0)=0, and $\left(d^{2}/dt^{2}\right)P\left(0\right)=-2\kappa \lambda$. As a consequence, a fourth-order closed differential equation with respect to time for the decoherence factor can be obtained:

(12) $$\begin{array}{c} \frac{d^{4}}{dt^{4}}D\left(t\right)+C_{3}\frac{d^{3}}{dt^{3}}D\left(t\right)+C_{2}\frac{d^{2}}{dt^{2}}D\left(t\right)+C_{1}\frac{d}{dt}D\left(t\right)+C_{0}D\left(t\right)=0, \end{array}$$

where the coefficients can be written as

(13) $$\begin{array}{c} C_{3}=2\kappa ,\;C_{2}=\kappa^{2}+\Omega^{2}+2\kappa \lambda +\nu^{2},\;C_{1}=2\kappa^{2}\lambda +2\kappa \nu^{2},\;C_{0}=\nu^{2}\left(\kappa^{2}+\Omega^{2}\right), \end{array}$$

and the initial conditions satisfy

(14) $$\begin{array}{c} D\left(0\right)=1,\;\frac{d}{dt}D\left(0\right)=-ia\nu ,\;\frac{d^{2}}{dt^{2}}D\left(0\right)=-\nu^{2},\;\frac{d^{3}}{dt^{3}}D\left(0\right)=-\nu^{2}-ia\nu . \end{array}$$

With the help of Laplace transformation taken over Equation (12), the decoherence factor D(t) can be analytically solved, in terms of the initial conditions in Equation (14), as

(15) $$\begin{array}{cc} D(t) & =L^{-1}\left[D\left(z\right)\right],\\ D(z) & =\frac{z^{3}+2\kappa z^{2}+\left(\kappa^{2}+\Omega^{2}+2\kappa \lambda \right)z+2\kappa^{2}\lambda +ia\nu \left(z^{2}+2\kappa z+\kappa^{2}+\Omega^{2}\right)}{z^{4}+2\kappa z^{3}+\left(\kappa^{2}+\Omega^{2}+2\kappa \lambda +\nu^{2}\right)z^{2}+2\kappa \left(\kappa \lambda +\nu^{2}\right)z+\left(\kappa^{2}+\Omega^{2}\right)\nu^{2}}. \end{array}$$

By means of the approach established in [68], the decoherence factor of the quantum system in time domain can be written as

(16) $$\begin{array}{cc} D(t) & =\sum_{j=1}^{n_{r}}\left[\frac{r_{j1}t^{e_{j}-1}}{(e_{j}-1)!}+\cdots +r_{je_{1}}\right]e^{a_{j}t}\\ \; & \;+\sum_{j=1}^{n_{c}}\left\{\left[\frac{c_{j1}t^{\epsilon_{j}-1}}{(\epsilon_{j}-1)!}+\cdots +c_{j\epsilon_{1}}\right]e^{b_{j}t}+\left[\frac{c_{j1}^{*}t^{\epsilon_{j}-1}}{(\epsilon_{j}-1)!}+\cdots +c_{j\epsilon_{1}}^{*}\right]e^{b_{j}^{*}t}\right\}, \end{array}$$

where $r_{jk}$ and $c_{jk}$ are the real and complex coefficients, which are respectively expressed as

(17) $$\begin{array}{cc} r_{jk} & =\frac{1}{(k-1)!}{\left\{\frac{d^{k-1}}{dz^{k-1}}\left[D\left(z\right){\left(z-a_{j}\right)}^{e_{j}}\right]\right\}}_{z=a_{j}},\\ c_{jk} & =\frac{1}{(k-1)!}{\left\{\frac{d^{k-1}}{dz^{k-1}}\left[D\left(z\right){\left(z-b_{j}\right)}^{\epsilon_{j}}\right]\right\}}_{z=b_{j}}. \end{array}$$

with $a_{j}$ and $b_{j}$ denoting the real and non-real roots of the denominator of D(z) in Equation (15) and the relation $\sum_{j}^{n_{r}}e_{j}+2\sum_{j}^{n_{c}}\epsilon_{j}=4$.

### 2.3. Comparisons with Previous Work

To compare this study in the present paper with that in previous work, we derived the expression of the decoherence factor in some special cases of the generalized RTN in the following.

We first considered the limit case that κ→+∞, namely the memoryless generalized RTN. Then, the expression of the decoherence factor under the influence of the generalized RTN in Equation (15) can be simplified as

(18) $$\begin{array}{cc} D(t) & =L^{-1}\left[D\left(z\right)\right],\;D\left(z\right)=\frac{z+2\lambda +ia\nu }{z^{2}+2\lambda z+\nu^{2}}. \end{array}$$

Consequently, the time domain decoherence factor D(t) can be expressed as

(19) $$D\left(t\right)=e^{-\lambda t}\left\{\begin{array}{cc} \left[\cosh\left(\chi t\right)+\frac{\lambda }{\chi }\sinh\left(\chi t\right)\right]+i\frac{a\nu }{\chi }\sinh\left(\chi t\right), & \nu <\lambda ,\\ \left(1+\lambda t\right)+ia\lambda t, & \nu =\lambda ,\\ \left[\cos\left(\chi t\right)+\frac{\lambda }{\chi }\sin\left(\chi t\right)\right]+i\frac{a\nu }{\chi }\sin\left(\chi t\right), & \nu >\lambda , \end{array}\right.$$

with $\chi =\sqrt{|\lambda^{2}-\nu^{2}|}$. This expression of the decoherence factor of the quantum system in Equation (19) recovers to that in [62]. Under the influence of the RTN only exhibiting the Markovian statistical property, two important regimes of dynamics have been distinguished: the weak-coupling (ν<λ) and the strong-coupling (ν>λ) regimes, and the dephasing dynamics displays the Markovian and non-Markovian characters in the two coupling regimes, respectively.

We now consider the case in which there is no environmental modulation of the memory effect with Ω=0, corresponding to an exponential-type memory kernel of the generalized RTN, namely $K\left(t-\tau \right)=\kappa e^{-\kappa (t-\tau )}$. In this case, the expression of the decoherence factor of the system in Equation (15) can be simplified as

(20) $$\begin{array}{cc} D(t) & =L^{-1}\left[D\left(z\right)\right],\;D\left(z\right)=\frac{z^{2}+\kappa z+2\kappa \lambda +ia\nu \left(z+\kappa \right)}{z^{3}+\kappa z^{2}+\left(2\kappa \lambda +\nu^{2}\right)z+\kappa \nu^{2}}. \end{array}$$

This expression of the decoherence factor under the influence of the generalized RTN with an exponential-type memory kernel in Equation (20) recovers to that in [61]. In this case of the RTN exhibiting the non-Markovian statistical property, the dephasing dynamics can also display a non-Markovian character even though the system–environment coupling is weak, and the boundary of the Markovian and non-Markovian dynamics regimes is determined by both the system–environment coupling and the memory effect of the generalized RTN [61,62].

## 3. Results and Discussion

In the following, we display the results of the dephasing dynamics of the quantum two-state system induced by nonequilibrium fluctuations in the environment exhibiting the generalized RTN statistical properties with a memory kernel of the modulatable-type. Our main focus is on the environmental effect of memory modulation on the quantum dynamical dephasing of the system under the influence of the generalized RTN in four types of regimes of the dephasing dynamics relying on the coupling ν of the environment and the decay rate of the environmental memory κ. In addition, we discuss the environmental effect of memory modulation on the conversion between Markovian dynamics and non-Markovian dynamics.

### 3.1. Dynamical Dephasing in Weak-Coupling Weak-Memory Regime

**Figure 1:**
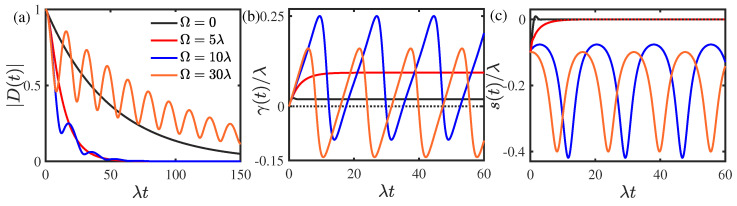
(Color online) The (**a**) decoherence factor |D(t)|, (**b**) decoherence rate γ(t), and (**c**) frequency shift s(t) as functions of time for different modulation frequencies Ω in the memory kernel in the weak-coupling weak-memory regime with the transition amplitude ν=0.2λ and memory decay rate κ=3λ. The initial non-stationary parameter of the environmental noise was set as a=0.5.

We first show the results of the dynamical dephasing in the weak-coupling weak-memory regime with the transition amplitude ν=0.2λ and memory decay rate κ=3λ. As shown in Figure 1a, the dephasing dynamics displays a Markovian character when there is no environmental effect of memory modulation, namely Ω=0. As the modulation frequency Ω increases, the dephasing dynamics is first enhanced and then suppressed, and the dynamical dephasing undergoes a conversion from a Markovian to a non-Markovian character related to a critical value $\Omega_{\mathrm{th}}$. When $\Omega >\Omega_{\mathrm{th}}$, the non-Markovian character begins to appear in the quantum dephasing dynamics of the system, and it becomes obvious with the increase of the modulation frequency. As depicted in Figure 1b, the decoherence rate γ(t) displays a monotonic increase to a constant value in a long time limit for small values of the modulation frequency, whereas it displays periodic oscillations for the modulation frequency greater than the critical value $\Omega_{\mathrm{th}}$. The decoherence rate γ(t) first increases with positive values and then begins to be negative in some time intervals as the modulation frequency Ω increases. When $\Omega >\Omega_{\mathrm{th}}$, the time intervals in which the decoherence rate is negative increase with the increase of the modulation frequency. The changes in the decoherence rate are in accordance with the character in the dynamical dephasing. Figure 1c displays the environmental effect of memory modulation on the energy re-normalization of the quantum system. Obviously, the frequency shift s(t) also shows a conversion from monotonic decay to disappearance in a long time limit to non-monotonically periodic oscillations with the increase of the modulation frequency.

### 3.2. Dynamical Dephasing in Weak-Coupling Strong-Memory Regime

**Figure 2:**
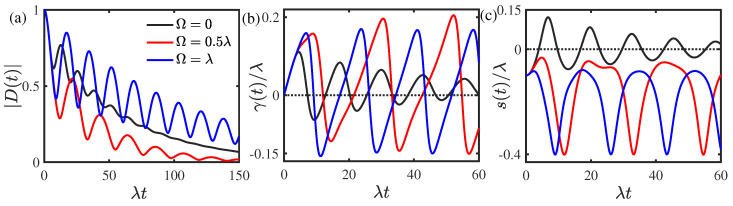
(Color online) The time-dependent (**a**) decoherence factor |D(t)|, (**b**) decoherence rate γ(t), and (**c**) frequency shift s(t) for different environmental modulation frequencies Ω in the memory kernel in the weak-coupling strong-memory regime with the transition amplitude ν=0.2λ and memory decay rate κ=0.1λ. The initial non-stationary parameter of the environmental noise was chosen as a=0.5.

We now discuss the case of the dynamical dephasing in the weak-coupling strong-memory regime with transition amplitude ν=0.2λ and memory decay rate κ=0.1λ. As displayed in Figure 2a, the dephasing dynamics always displays a non-Markovian character even though the system–environment coupling is weak, which is mainly a result of the strong memory effect of the generalized RTN. As the modulation frequency Ω increases, the dephasing dynamics of the system is first increased and then reduced. Meanwhile, the non-Markovian character in the dephasing dynamics becomes prominent. As depicted in Figure 2b, the decoherence rate γ(t) decays monotonically for small modulation frequencies, whereas it displays non-monotonic periodic oscillations for large modulation frequencies. With the increase of the modulation frequency, the time intervals for which the decoherence rate takes positive values first increase and then decrease, whereas the time intervals in which the decoherence rate is negative increase. The character in the decoherence rate is consistent with that in the dephasing dynamics of the quantum system. As depicted in Figure 2c, the frequency shift s(t) shows a non-monotonic decay and vanishes in a long time limit for small modulation frequencies, whereas it shows non-monotonically periodic oscillations when the modulation frequency is greater than some values.

### 3.3. Dynamical Dephasing in Strong-Coupling Weak-Memory Regime

**Figure 3:**
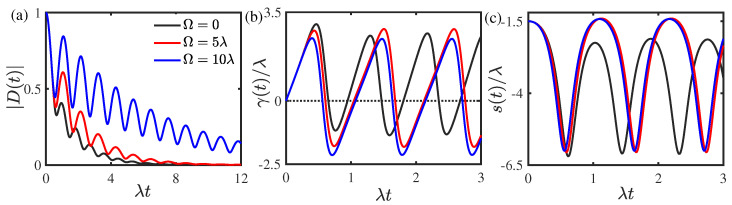
(Color online) The (**a**) decoherence factor |D(t)|, (**b**) decoherence rate γ(t), and (**c**) frequency shift s(t) as functions of time for different modulation frequencies Ω in the memory kernel in the strong-coupling weak-memory regime with the transition amplitude ν=3λ and memory decay rate κ=4λ. The initial non-stationary parameter of the environmental noise was set as a=0.5.

In this subsection, we discuss the case of the dynamical dephasing in the strong-coupling weak-memory regime with transition amplitude ν=3λ and memory decay rate κ=4λ. As depicted in Figure 3a, the dephasing dynamics of the system always shows a non-Markovian character arising from the strong coupling with the environment. As the modulation frequency Ω increases, the dynamical dephasing is suppressed and the non-Markovian character in the dephasing dynamics of the quantum system becomes obvious. As depicted in Figure 3b, the decoherence rate γ(t) always shows periodic oscillations with discrete zeros. The time intervals in which the decoherence rate is negative increase as the modulation frequency increases. As displayed in Figure 3c, the frequency shift s(t) displays non-monotonic periodic oscillations.

### 3.4. Dynamical Dephasing in Strong-Coupling Strong-Memory Regime

**Figure 4:**
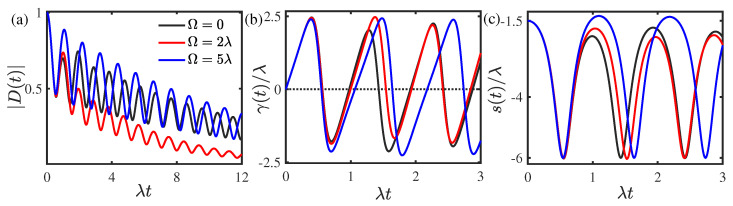
(Color online) The time-dependent (**a**) decoherence factor |D(t)|, (**b**) decoherence rate γ(t), and (**c**) frequency shift s(t) for different modulation frequencies Ω in the memory kernel in the strong-coupling strong-memory regime with the transition amplitude ν=3λ and memory decay rate κ=1λ. The initial non-stationary parameter of the environmental noise was chosen as a=0.5.

Finally, we show the results of the dynamical dephasing in the strong-coupling strong-memory regime with ν=3λ and κ=λ. As displayed in Figure 4a, the dephasing dynamics always show a non-Markovian character owing to both the strong interaction with the environment and the strong memory effect of the generalized RTN. With the increase of the modulation frequency Ω, the dynamical dephasing of the quantum system and the non-Markovian character in the dephasing dynamics is first suppressed and then enhanced. As depicted in Figure 4b, the decoherence rate γ(t) always shows periodic oscillations with discrete zeros. The time intervals that the decoherence rate is negative first decrease and then increase as the modulation frequency increases. As shown in Figure 4c, the frequency shift s(t) displays non-monotonic periodic oscillations, which is similar to the case in the strong-coupling weak-memory regime.

### 3.5. Conversion between Markovian and Non-Markovian Characters in Dephasing Dynamics

According to the above results discussed in four types of dynamics regimes, we can see that the dynamical dephasing of the quantum system and the non-Markovian character exhibited in the dephasing dynamics under the influence of the generalized RTN can be effectively modulated via the environmental memory kernel. It is worth noting that we can encounter a non-Markovian character in the dephasing dynamics by controlling the modulation frequency of the environmental memory kernel in the weak-coupling weak-memory regime. Under the influence of the environmental effect of memory modulation, the boundary of the Markovian and non-Markovian characters in the dynamical dephasing closely depends on the modulation frequency of the generalized RTN. In the following, we show the conversion from the Markovian to the non-Markovian character in the quantum dephasing dynamics of the system in the parameter space of ν∼κ for different environmental modulation frequencies Ω.

**Figure 5:**
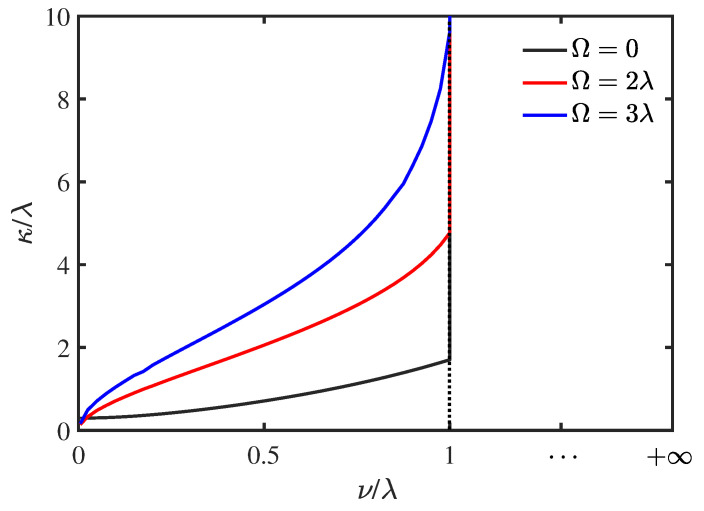
(Color online) Phase diagram of the conversion from the Markovian to the non-Markovian character in the dephasing dynamics of the quantum system for different environmental modulation frequencies Ω. The upper-left and lower-right regions of the curves are the Markovian and non-Markovian dynamical regions, respectively. The black dotted line stands for the dynamical boundary of the conversion induced by the standard RTN, namely the boundary between weak and strong couplings.

Figure 5 shows the phase diagram of Markovian and non-Markovian dynamical conversion in the ν∼κ space in terms of the non-Markovianity defined in Equation (4) in the presence of different environmental modulation effects. In the strong-coupling regime (ν>λ), the dephasing dynamics of the quantum system always displays a non-Markovian character (N>0), whereas it undergoes a conversion from a Markovian (N=0) to a non-Markovian (N>0) character with the increase of the transition amplitude ν in the weak-coupling regime (ν<λ). Furthermore, for a given coupling strength ν, the larger the modulation frequency Ω is, the larger the critical value of the memory decay rate $\kappa_{\mathrm{th}}$ of the conversion for the dynamical boundary is. For example, for ν=0.8λ, the critical values are $\kappa_{\mathrm{th}}=1.227\lambda$ for Ω=0, $\kappa_{\mathrm{th}}=3.263\lambda$ for Ω=2λ, and $\kappa_{\mathrm{th}}=5.106\lambda$ for Ω=3λ, respectively. That is, the non-Markovian region of dynamical dephasing increases as the modulation frequency Ω increases. It is worth mentioning that we can realize the conversion of the Markovian and non-Markovian characters in the dephasing dynamics by changing the environmental modulation frequency in the weak-coupling weak-memory regime. However, in the other three dynamics regimes, we cannot realize the conversion from the non-Markovian character (N>0) in the dephasing dynamics with no environmental modulation, namely Ω=0, to the Markovian character (N=0) in the dynamical dephasing by changing the modulation frequency in the environmental memory kernel.

## 4. Conclusions

We performed a theoretical study of the quantum dynamical dephasing of a two-state system that interacts with a classical environment, which displays non-equilibrium fluctuations. Under the influence of the environmental fluctuations governed by a generalized RTN process with a modulatable-type memory kernel, we derived a closed time differential equation of fourth-order for the decoherence factor of the system and obtained the analytical solution of the decoherence factor exactly. For some special limit cases of the environmental memory kernel, the expression of the decoherence factor of the system can be simplified as the ones that have been derived in previous work. We analyzed the environmental effect of memory modulation in the dephasing dynamics in four types of regimes, respectively. The results showed that the dynamical dephasing of the system and the non-Markovian character exhibited in the dephasing dynamics can be effectively modulated via the environmental memory kernel. It is worth mentioning that we can encounter non-Markovian characters by changing the modulation frequency of the environmental memory kernel in the weak-coupling weak-memory regime, which have rarely been reported in previous studies. We also plotted the phase diagram to investigate the environmental influence of the memory modulation on the Markovian and non-Markovian dynamical transition in the parameter space in terms of the system–environment coupling and the memory effect of the generalized RTN. The results showed that, in the strong-coupling regime, the dynamical dephasing of the quantum system always displays a non-Markovian character, whereas in the weak-coupling regime, it suffers from a conversion from a Markovian to a non-Markovian character, for which the boundary is determined by the combined effects of the system–environment coupling, the decay rate in the environmental memory kernel, and the environmental modulation frequency of the memory kernel.

## Data Availability

Not applicable.

## Abbreviations

The following abbreviation is used in this manuscript:

RTN	Random telegraph noise
